# Effect of the finite speed of light in ionization of extended molecular systems

**DOI:** 10.1038/s41598-021-00818-1

**Published:** 2021-11-02

**Authors:** I. A. Ivanov, Anatoli S. Kheifets, Kyung Taec Kim

**Affiliations:** 1 Center for Relativistic Laser Science, Institute for Basic Science, Gwangju, 61005 Korea; 2 Research School of Physics, The Australian National University, Canberra, ACT 2601 Australia; 3 Department of Physics and Photon Science, GIST, Gwangju, 61005 Korea

**Keywords:** Atomic and molecular interactions with photons, Attosecond science

## Abstract

We study propagation effects due to the finite speed of light in ionization of extended molecular systems. We present a general quantitative theory of these effects and show under which conditions such effects should appear. The finite speed of light propagation effects are encoded in the non-dipole terms of the time-dependent Shrödinger equation and display themselves in the photoelectron momentum distribution projected on the molecular axis. Our numerical modeling for the $\hbox {H}_{2}^{+}$ molecular ion and the $\hbox {Ne}_2$ dimer shows that the finite light propagation time from one atomic center to another can be accurately determined in a table top laser experiment which is much more readily accessible than the ground breaking synchrotron measurement by Grundmann et al. (Science 370:339, 2020).

## Introduction

Every quantum system evolves on its characteristic time scale which varies widely between molecules (femtoseconds—$10^{-15}\ \hbox {s}$)^1^, atoms (attoseconds—$10^{-18}\ \hbox {s}$)^2^ and nuclei (zeptoseconds—$10^{-21}\ \hbox {s}$)^3^. A crossover between these time scales is very rare in nature. It was therefore quite unexpected to discover an ionization process in the hydrogen molecule that evolved on a zeptosecond time scale^4^. An explanation of this phenomenon appeared to be quite simple. While it takes tens of attoseconds for an electron to trespass the $\hbox {H}_2$ molecule, the incoming light wave sweeps from one molecular end to another orders of magnitude faster. This results in one of the constituent hydrogen atoms getting ionized a fraction of the attosecond sooner than its counterpart. Such a tiny ionization delay manifests itself quite noticeably in the two-slit electron interference that the $\hbox {H}_2$ molecule readily displays^5^. To discover a zeptosecond delay in molecular photoionization in^4^ the authors needed to deploy an extremely bright synchrotron source of highly energetic photons^6^. We demonstrate that even a table top laser experiment is capable of detecting a similar effect making it much more readily affordable.

In this work we present a general quantitative theory of the delay due to the finite speed of light propagation and we show under which conditions such effects should manifest themselves. In our numerical demonstrations, we consider the $\hbox {H}_{2}^{+}$ molecular ion and the $\hbox {Ne}_2$ dimer. The $\hbox {H}_{2}^{+}$ molecular ion has been scrutinized since the early days of quantum mechanics^7^ and was recently used as a model for the study of the interference effects in photon-momentum transfer for the process of molecular ionization^8^. Photoionization studies of $\hbox {Ne}_2$ is a novelty^9^. We subject both targets to an attosecond laser pulse that can be readily produced in high-order harmonics generation sources^10,11^. The photoelectron flux encodes the timing information about the ionization process. This flux is reconstructed by solving the laser-driven time-dependent Schrödinger equation (TDSE). The numerical results obtained for $\hbox {H}_{2}^{+}$ using the TDSE can be interpreted in a transparent qualitative way by considering a very simple heuristic tight-binding model (TBM). This gives us a tool for understanding the time delay caused by the finite speed of light propagation. We apply this tool to $\hbox {H}_{2}^{+}$ and $\hbox {Ne}_2$ and find the time delay of a fraction of attosecond that depends sensitively on the orientation of the molecular axis relative to the propagation and polarization directions.

Atomic units (a.u.) are used throughout the paper with the electron charge *e*, mass *m* and the Planck constant $\hbar$ all set to unity $m=e=\hbar =1$. The speed of light in these system of units is $c\approx 137.036$ a.u.

## Results

### Theoretical model

Our approach is based on the numerical solution of the three-dimensional TDSE

1 $$\begin{aligned} i \partial \Psi (\varvec{r},t) / \partial t=\left[ \hat{H}_{\mathrm{mol}} + \hat{H}_{\mathrm{int}}(t)\right] \Psi (\varvec{r},t) \ , \end{aligned}$$

where $\hat{H}_{\mathrm{mol}}$ is the field-free one-electron Hamiltonian and $\hat{H}_{\mathrm{int}}(t)$ describes the field-target interaction. We consider the hydrogen molecule ion H$_2^+$ in the ground state as a target. The ion interacts with a short (total duration of four optical cycles) pulse with the base frequency $\omega =4.04$ a.u. (photon energy of 110 eV) with the peak field strength $E_0=0.1$ a.u. (intensity of $3.51\times 10^{14}\ \hbox {W}/\hbox {cm}^2$). The pulse described by the vector potential $\varvec{A}(t-x/c)$ propagates in the positive *x*-direction and is polarized in the *z*-direction.

We apply the procedure previously used in^12^ to study non-dipole effects in atomic photoionization. The leading order relativistic corrections to $\hat{H}_{\mathrm{int}}(t)$ come from the linear term of the expansion of $\varvec{A}(t-x/c)$ in powers of $c^{-1}$. Such an expansion leads to the following Hamiltonian describing the target-field interaction:

2 $$\begin{aligned} \hat{H}_{\mathrm{int}}(\varvec{r},t) = \hat{p}_z A(t)+ {\hat{p}_z x E(t)\over c}+ {A(t)E(t)x\over c} \ . \end{aligned}$$

More detailed description of the derivation of the Hamiltonian (2) and details of the numerical procedure we used to solve the TDSE (1) can be found in the “Methods” section. The interaction Hamiltonian (2) can be related by a gauge transformation to the Kramers-Henneberger Hamiltonian used in^13^.

An additional source of relativistic corrections in $\hat{H}_{\mathrm{int}}$ is the interaction of the magnetic field of the pulse with the electron spin. These corrections cannot be obtained by a simple generalization of the minimal coupling Hamiltonian. To obtain them, one needs to consider systematically the transition of the Dirac equation to the non-relativistic limit^14^. The inclusion of the electron spin, however, is not necessary for the present study where we keep only the $c^{-1}$ terms as was done, for instance, in^12,15^. The Breit–Pauli relativistic corrections to $\hat{H}_{\mathrm{mol}}$, such as the effects of the relativistic kinematics, spin––orbit interaction and the so-called Darwin term, are all of the order of $c^{-2}$ and can also be safely omitted^16^ .

For a weak electromagnetic field that we employ, we can also obtain an analytical expression for the ionization amplitude by using the perturbation theory (PT) and treating the operator (2) as a perturbation:

3 $$\begin{aligned} a_{\varvec{p}}= -i\int _{-\infty }^{+\infty } \langle \phi ^{-}_{\varvec{p}}| \hat{H}_{\mathrm{int}}(\tau )|\phi _0\rangle e^{i(E_{\varvec{p}} -\varepsilon _0)\tau }\ d\tau \equiv a^{(0)}_{\varvec{p}} + a^{(1)}_{\varvec{p}} \ . \end{aligned}$$

Here $\phi _0$ and $\phi ^-_{\varvec{p}}$ are the initial and final molecular states with the corresponding energies $\varepsilon _0$ and $E_{\varvec{p}}$.

We split the ionization amplitude (3) into the nonrelativistic part $a^{(0)}_{\varvec{p}}$ and the first order relativistic correction $a^{(1)}_{\varvec{p}}$. By introducing the Fourier transforms $A(t) = (2\pi )^{-1} \int a(\Omega ) e^{-i t\Omega }\ d\Omega$ and $A^2(t) = (2\pi )^{-1} \int b(\Omega ) e^{-i t\Omega }\ d\Omega$ we obtain for these amplitudes:

4 $$\begin{aligned} a^{(0)}_{\varvec{p}}&= -i a(\Omega ) \langle \phi ^-_{\varvec{p}}|\hat{p}_z|\phi _0 \rangle \ \ \ , \ \ \Omega = E_{\varvec{p}}- \varepsilon _0 \end{aligned}$$

5 $$\begin{aligned} a^{(1)}_{\varvec{p}}&= \Omega c^{-1} \left( a(\Omega )\langle \phi ^-_{\varvec{p}}| \hat{p}_z x|\phi _0 \rangle \ + b(\Omega ) \langle \phi ^-_{\varvec{p}}|x |\phi _0 \rangle /2 \right) \nonumber \\&\approx \Omega c^{-1}a(\Omega ) \langle \phi ^-_{\varvec{p}}| \hat{p}_z x|\phi _0 \rangle \ . \end{aligned}$$

The second term on the right hand side of Eq. (5) can be neglected because the ratio of the second term and the first term in this expression is approximately $b(\Omega )/(a(\Omega ) p)$, where *p* is the typical value of the momentum of the ionized electron. For the pulse parameters that we consider, this value can be estimated as $E_0/(\omega p)\approx 0.01$.

It can be seen from Eq. (5) that the relativistic correction $a^{(1)}_{\varvec{p}}$ vanishes in certain cases. In particular, it is zero for an axially symmetric system with the field-free Hamiltonian that is invariant under rotations about the *z*-axis. Indeed, for such systems the scattering state $\phi ^-_{\varvec{p}}(\varvec{r})$ is a function of the arguments $z, p_z, \rho , p_{\rho }$ and ${\varvec{\rho }}\cdot \varvec{p}_{\rho }$, where *z*, $p_z$ and ${\varvec{\rho }}$, $\varvec{p}_{\rho }$ are the components of the $\varvec{r}$ and $\varvec{p}$ vectors parallel and perpendicular, respectively, to the *z*- axis of the coordinate system that we employ. For an axially symmetric system, the ground state wave function $\phi _0$ in Eq. (5) (which we assume to be non-degenerate) is invariant under rotations about the *z*-axis. We obtain then from Eq. (5): $\displaystyle a^{(1)}(p_x,p_y,p_z)=-a^{(1)}(-p_x,p_y,p_z)$. Therefore the amplitude $a^{(1)}_{\varvec{p}}$ is zero in the plane $p_x=0$ which encompasses the most important region of the momenta near the maximum of the momentum distribution.

The effect of the propagation correction (5) can be most easily illustrated within the tight-binding model (TBM) in which the ground state of $\hbox {H}_{2}^{+}$and $\hbox {Ne}_2$is represented by a Heitler-London wave function:

6 $$\begin{aligned} \phi _0(\varvec{r})= [\phi (\varvec{r}-{\varvec{R}/ 2}) + \phi (\varvec{r}+{\varvec{R}/ 2})]/\sqrt{2} \ . \end{aligned}$$

In the TBM, the overlap of the two terms is small and $\phi (\varvec{r})$ is represented by a spherically symmetric atomic-like state. Under these conditions, and by employing the Born approximation for the scattering state $\phi ^-_{\varvec{p}}$, one can show (the formal derivation is given in the section “Methods”) that Eq. (4) and Eq. (5) lead to the following expression for the ionization amplitude:

7 $$\begin{aligned} a_{\varvec{p}}= a^{(0)}_{\varvec{p}} + a^{(1)}_{\varvec{p}} \approx a_d(\varvec{p}) \sqrt{2} \cos {\left( {\varvec{p}\cdot {\varvec{R}}/ 2} +\delta \right) } \ , \end{aligned}$$

where $\delta =- {\varvec{\kappa }}\cdot {\varvec{R}}/2$ and ${\varvec{\kappa }}=\Omega c^{-1}\varvec{e}_x$ is the photon momentum, and $a_d(\varvec{p})$ is the dipole amplitude which we would obtain if electrons were emitted from the state described by the single-center initial wave-function $\phi (\varvec{r})$ centered at the origin. It is this phase factor $\delta$ in Eq. (7) that is responsible for the modified interference pattern observed and decoded in^4^.

Appearance of this additional phase in Eq. (7) is due to an extra propagation time of the light wave from one end of the molecule to the other. This interpretation becomes yet more transparent if we use coordinate representation for the part of the wave-function describing the ionized wave packet:

8 $$\begin{aligned} \Psi _{\mathrm{ion}}(\varvec{r},t)= \int a_{\varvec{p}} \phi ^{-}_{\varvec{p}}(\varvec{r}) e^{-iE_{\varvec{p}}t}\ d\varvec{p}\ . \end{aligned}$$

Here $\phi ^{-}_{\varvec{p}}(\varvec{r})$ are the molecular scattering states. To evaluate this integral in the limit $t\rightarrow \infty$, we rely on the saddle-point method (SPM) that is commonly used in description of ionization^17,18^ or scattering^19^ processes. By writing $a_d(\varvec{p})=|a_d(\varvec{p})|e^{i\eta (\varvec{p})}$ in Eq. (7) and employing the SPM, we obtain from Eq. (8) (the details of the derivation are given in the section “Methods”):

9 $$\begin{aligned} \mathop {\lim }\limits_{{t \to \infty }} \Psi _{{{\text{ion}}}} (\user2{r},t) & \simeq \left( {z + \frac{{R_{z} }}{2}} \right)\exp \left\{ {i\frac{{\left( {\user2{r} + \frac{\user2{R}}{2}} \right)^{2} \left( {\frac{t}{2} - \tau - \tau _{1} } \right)}}{{(t - \tau - \tau _{1} )^{2} }} + i\delta } \right\}G\left( {\left| {\user2{r} + \frac{\user2{R}}{2}} \right| - p_{0} (t - \tau - \tau _{1} )} \right) \\ & \quad + \left( {z - \frac{{R_{z} }}{2}} \right)\exp \left\{ {i\frac{{\left( {\user2{r} - \frac{\user2{R}}{2}} \right)^{2} \left( {\frac{t}{2} - \tau + \tau _{1} } \right)}}{{(t - \tau + \tau _{1} )^{2} }} - i\delta } \right\}G\left( {\left| {\user2{r} - \frac{\user2{R}}{2}} \right| - p_{0} (t - \tau + \tau _{1} )} \right). \\ \end{aligned}$$

The value $p=p_0$ in Eq. (9) is the momentum values satisfying energy conservation $p_0^2/2= \varepsilon _0+ \omega$, $\displaystyle \tau = {\partial \eta / \partial E}$ is the usual Wigner photoemission time-delay^20^ and $\tau _1 ={\partial \delta / \partial E}= -R_x/(2c)$ with $\delta =-{\varvec{\kappa }}\cdot {\varvec{R}}/2$ from Eq. (7). The $\tau _1$ term represents an additional time-delay that it takes for the light pulse to cover the distance $R_x= {\varvec{R}}\cdot \varvec{e}_x$. The function *G*(*u*) in Eq. (9) is sharply peaked near the origin. Therefore the two terms in Eq. (9) describe two wave packets emitted from the two atomic centers $\varvec{r}=-\varvec{R}/2$ and $\varvec{r}=\varvec{R}/2$ at the times $\tau + \tau _1$ and $\tau -\tau _1$, respectively.

For transparency of derivation, we omitted in Eq. (9) the Coulomb terms which would only add slowly varying (logarithmic) corrections in the arguments of *G*(*u*)^17,18^. These additional logarithmic terms are the same for the two wave packets and they would therefore cancel in the time delay difference between these wave packets.

We note that the dipole amplitude $a_d(\varvec{p})$ in the Eq. (7) is essentially a product of two factors, the factor $p_z$ responsible for the angular dependence of the amplitude and the Gaussian factor $\exp {\left\{ -b(p-p_0)^2\right\} }$ representing the energy conservation $p_0^2/2= \varepsilon _0+ \omega$. Such a Gaussian representation of the ionized wave packets emitted in the single-center problems is often used in studying temporal dynamics of atomic ionization^17,18^. The parameter *b* determines the width of the wave packet and depends on the pulse parameters (more details are given in the section “Methods”). By employing the Gaussian ansatz for $a_d(\varvec{p})$ we finally obtain from Eq. (7):

10 $$\begin{aligned} a_{\varvec{p}} = A \exp {\left\{ -b(p-p_0)^2\right\} } p_z \cos {\left( {\varvec{p}\cdot {\varvec{R}}/ 2} +\delta \right) } \ . \end{aligned}$$

**Figure 1:**
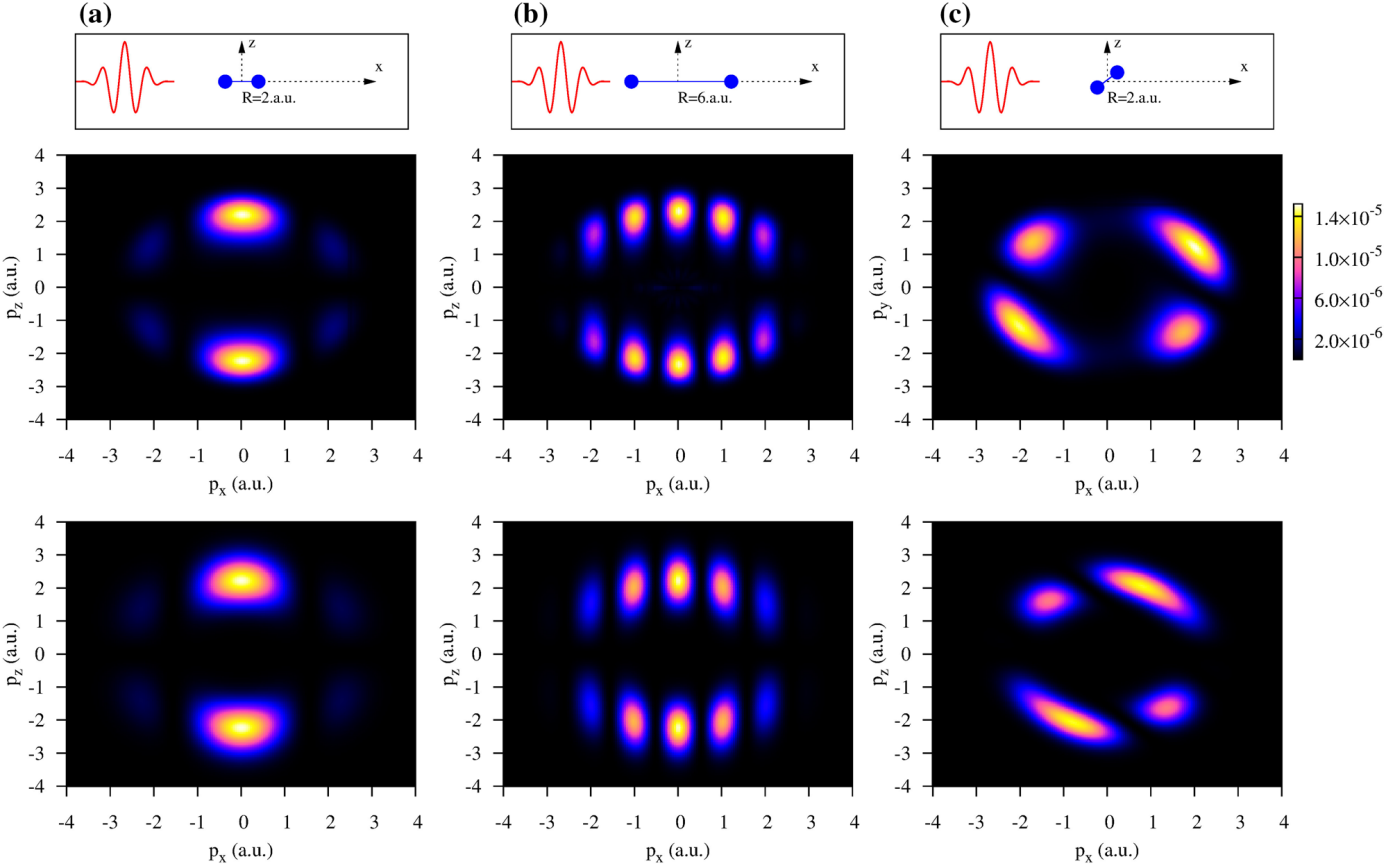
(Color online) The photoelectron momentum distributions projected on the $(p_x,p_y)$-plane for different orientations and inter-nuclear distances of $\hbox {H}_{2}^{+}$illustrated in the top row of panels. The middle row of panels displays the numerical TDSE results while the bottom row exhibits the corresponding TBM results obtained using Eq. (7).

We use Eq. (10) to evaluate the photoelectron emission pattern and to compare it with the fully *ab initio* TDSE calculations for various orientations and different inter-nuclear distances of $\hbox {H}_{2}^{+}$. This comparison is presented in Fig. 1. The top row of panels illustrates the geometry of the ionization process. It is assumed that the molecular axis is confined to the *xz*-plane where the propagation and polarization vectors of the pulse belong, making an angle $\theta$ with the propagation direction. In (a-b), $\theta =0$ while in (c) $\theta =\pi /4$. The photoelectron momentum distribution is projected on the *xz* plane and computed as $P(p_x,p_z)=\int |a_{\varvec{p}}|^2\ dp_y$ with the amplitude $a_{\varvec{p}}$ obtained by projecting the TDSE solution on the set of the scattering states of $\hbox {H}_{2}^{+}$. The number and location of the bright spots in Fig. 1 reflect a simple two-center interference pattern governed by the cosine term in Eq. (10).

## Discussion

Comparison of the TDSE calculations (the middle row of panels in Fig. 1) and results based on Eq. (10) (the bottom row of panels) shows that the TBM reproduces the spectra adequately for the geometries that we consider. The TBM certainly has some limitations if we demand an accurate global representation of the spectra. For instance, for the case of the non-collinear orientation of the polarization vector and the molecular axis shown in Fig. 1, the *ab initio* TDSE and the TBM spectra exhibit some differences. One could improve, in principle, the visual agreement of the TDSE and the TBM results in this case by adjusting the value of the fitting parameter *b* in Eq. (10). The value of this parameter affects the tilt of the TBM distribution with respect to the *x*-axis, so we can rotate the TBM spectrum by varying this parameter. Achieving a good global agreement between the TDSE and TBM is, however, not needed for the purposes of the present work. For the procedure of the extraction of the relativistic delays that we describe below to work, we need Eq. (10) to accurately describe the spectra locally, in the vicinity of a maximum. We will see below that this lesser goal can be achieved. We will, therefore, analyze and interpret our TDSE results using the transparent TBM that is equally applicable to both $\hbox {H}_{2}^{+}$ and $\hbox {Ne}_2$. We will focus on the photoelectron momentum distribution projected on the (*xz*) plane and integrated over the momentum component $p_{\perp }$ which is perpendicular to the molecular axis. Such a momentum distribution is a function of the momentum component $p_{||}$ which is parallel to the molecular axis. By employing the Gaussian ansatz (10) we obtain:

11 $$\begin{aligned} P(p_{||})= \int P(\varvec{p})\ dp_y dp_{\perp } \approx B \exp {\left\{ -C p_{||}^2\right\} } \cos ^2{\left( {p_{||}R\over 2} +\delta \right) } \ . \end{aligned}$$

**Figure 2:**
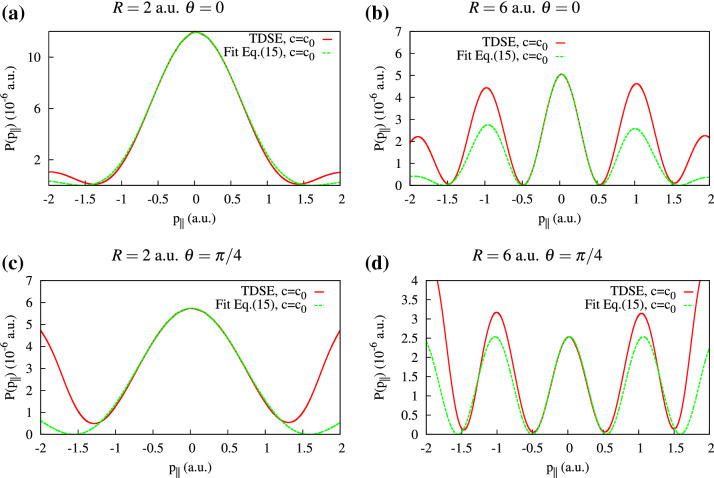
(Color online) The photoelectron momentum distribution projected on the molecular axis for various geometries and internuclear distances. The fit based on Eq. (11) and TDSE calculations are plotted with the solid and dotted lines, respectively.

We use the analytical expression (11) with adjustable parameters *B*, *C* and $\delta$ to fit $P(p_\Vert )$ obtained from the numerical TDSE calculations. The accuracy of the fitting procedure is illustrated in Fig. 2 where we display $P(p_\Vert )$ for various geometries and internuclear distances. In the cases of $\theta =0$ $P(p_\Vert )$ is simply the projection of the 2D momentum distribution on the horizontal axis. We use the interval of the $p_\Vert \in (-0.5,0.5)$ for the fitting procedure. As is seen in Fig. 2, the analytical fit with Eq. (11) represents the central maximum of the TDSE calculations with the corresponding *R* value quite accurately. This allows us to extract the phase shift $\delta$ accumulated due to the finite speed of light propagation.

**Figure 3:**
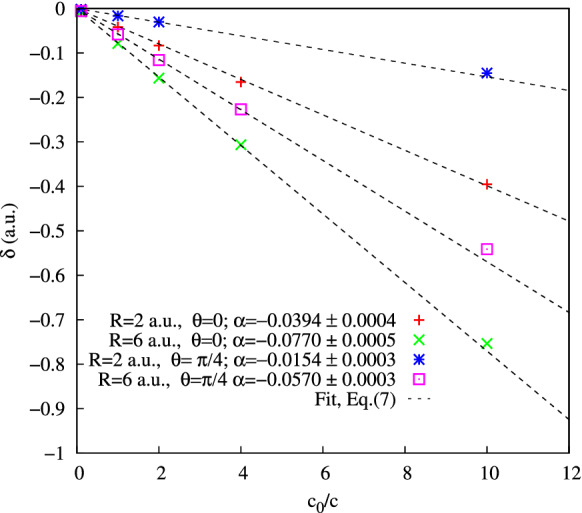
(Color online) Parameter $\delta$ in Eq. (11) for different geometries and internuclear distances.

In Fig. 3 we display thus extracted phase shifts $\delta$ for the molecular orientations and internuclear distances illustrated in Fig. 2. To enhance the relativistic effects, we artificially decrease the speed of light in the TDSE calculations from its physical value $c_0=137.036$ a.u. down to $c=c_0/10$. According to TBM, the phase shift should scale linearly as $\delta = \alpha c_0/c$ with the slope $\alpha = -R_x\kappa /2$. The predicted linear scaling is confirmed very accurately by the numerical values shown in Fig. 3 with only a small error margin. The time delay values corresponding to the physical speed of light $c_0$ are shown in Table 1 in comparison with the estimate $\Delta t= 2R_x/c_0$ provided by the TBM. Agreement of the results can be deemed quite satisfactory given the relative simplicity of TBM. More importantly, the linear dependence of the TDSE results on the parameter $c_0/c$ clearly demonstrates existence of the finite speed of light effect in ionization of diatomic molecules.

**Table 1:** Propagation delay for different geometries and internuclear distances.

R (a.u.)	$\theta$	Fit (as)	$R_x/c$ (as)
2	0	0.46	0.35
2	$\pi /4$	0.18	0.24
6	0	0.90	1.05
6	$\pi /4$	0.67	0.72

## Conclusions

In conclusion, our work was motivated by the synchrotron based experiment^4^ which discovered a zeptosecond time delay in photoionization of the $\hbox {H}_2$ molecule. We aimed to demonstrate that a similar delay, that is caused by the finite speed of light propagation from one constituent atom to another, can be detected in a much more accessible table top laser settings. In doing so, we developed a general theory of the finite speed of light propagation effects in ionization of extended systems. As a simple case study, we considered the $\hbox {H}_{2}^{+}$ molecular ion interacting with a short laser pulse. This target affords a very accurate numerical treatment within the time-dependent Schrödinger equation. At the same time, an heuristic tight-binding model employing the Heitler-London molecular ground state produces very similar results. TBM can be easily adopted to the $\hbox {Ne}_2$ by a simple increase of the inter-atomic distance to $R\simeq 6$ a.u. Notably, the corresponding interference pattern displayed in Fig. 1b) is very similar to that obtained in the experiment^9^ and the earlier SPM modeling^21^.

Our simulations demonstrate that the speed of light delay in ionization of diatomic molecular targets can be decoded from the photoelectron momentum distribution projected on the molecular axis. This method has a clear advantage over the earlier synchrotron measurement based on decoding the 2D interference pattern^4^. Indeed, the bright interference spots have a finite angular width. To detect their shift due to the finite speed of light requires a sufficiently large photon momentum that should not be vanishingly small in comparison with the photoelectron momentum. This in turn requires very high photon energy (800 eV in^4^). Fitting of a one-dimensional momentum distribution produces significantly reduced error bars. Thus smaller values of the photon energy of the order of 100 eV can be used. Driving pulses with the parameters employed in our simulations (110 eV photon energy, intensity of $3.51 \times 10^{14}\ \hbox {W}/\hbox {cm}^2$) can be obtained by using relativistic high harmonic generation. It was shown^22^ that even the Schwinger intensity ($10^{29}\ \hbox {W}/\hbox {cm}^2$) can be reached by reflecting the currently available PW laser beam with intensity of $10^{22}\ \hbox {W}/\hbox {cm}^2$ on a plasma mirror. Intensities of the order of $10^{21}\ \hbox {W}/\hbox {cm}^2$ can be reached by using a table-top 100 TW system^23^. In addition, by projecting the momentum distribution on the molecular axis, we increase the count rate and improve statistics of the measurement. Hence the photon flux can be significantly reduced. This reduction of both the photon flux and energy makes the proposed method much more readily accessible in desk-top conventional laser settings. This we hope will stimulate further speed of light delay determinations in diatomic molecules and other extended systems. Incidentally, the finite *c* delay measured in $\hbox {H}_2$ deviates from accurate theoretical modeling^24^. The proposed novel technique may shed an additional light on the nature of this disagreement.

## Methods

### Leading order relativistic corrections to the interaction Hamiltonian and details of the numerical procedure

The leading order relativistic corrections to the non-relativistic Hamiltonian describing the target-field interaction arise from the vector potential being a traveling wave $\displaystyle \varvec{A}(\eta )$ with $\eta = t-x/c$ (we assume that the pulse propagates in the positive *x*-direction and is polarized in *z*-direction). The leading relativistic correction to non-relativistic interaction Hamiltonian can be obtained by substituting this expression for the vector potential into the standard minimal coupling Hamiltonian $\displaystyle \hat{H}_{\mathrm{min}}= {1\over 2} (\hat{\varvec{p}} + \varvec{e}_z A(x,t))^2$^16^, and keeping the terms linear in $c^{-1}$. One arrives then at the expression for the interaction Hamiltonian^12^:

12 $$\begin{aligned} \hat{H}^{\mathrm{r}}_{\mathrm{int}}(\varvec{r},t) = \hat{p}_z A(t) +{\hat{p}_z x E(t)\over c}+ {A(t)E(t)x\over c} + {A^2(t)\over 2}\ , \end{aligned}$$

where *A*(*t*) no longer depends on the coordinates, and $\displaystyle E(t)=-{\partial A(t)\over \partial t}$ is the electric field of the pulse. The last term on the r.h.s. of the Eq. (12) is a function of time only, and can therefore be removed by a unitary transformation of the wave-function.

To solve the TDSE with the interaction Hamiltonian (12) we use the procedure that we employed previously for the solution of the non-relativistic TDSE^25–27^. Solution of the TDSE is represented as an expansion:

13 $$\begin{aligned} \Psi ({\varvec{r}},t)= \sum \limits _{l,m}^{l_{\mathrm{max}}} f_{lm}(r,t) Y_{lm}(\theta ) \ , \end{aligned}$$

where $Y_{lm}(\theta )$ are the spherical harmonics. The radial variable is treated by discretizing the TDSE on a grid with the step-size $\delta r=0.1$ a.u. in a box of the size $R_{\mathrm{max}}$. The values of the parameters $R_{\mathrm{max}}$ and $l_{\mathrm{max}}$ in Eq. (13) were chosen (after the necessary convergence checks) as: $l_{\mathrm{max}}=10$, $R_{\mathrm{max}}=400$ a.u.

For the vector potential in Eq. (12) we used the form:

14 $$\begin{aligned} A(t)= -{E_0\over \omega } \sin ^2{\pi t\over T_1}\sin {\omega t} \ , \end{aligned}$$

with $\omega =4.04$ a.u. (photon energy of 110 eV), peak field strength $E_0=0.1$ a.u. (intensity of $3.51\times 10^{14}$ W/cm$^2$), and total duration $T_1=4T$, where $T=2\pi /\omega$- optical cycle corresponding to the base frequency $\omega$.

The potential energy in the field-free H$_2^+$ Hamiltonian:

15 $$\begin{aligned} \hat{H}_{\mathrm{mol}}= {\hat{\varvec{p}}^2\over 2} -{1\over |\varvec{r}-{\varvec{R}/2}|} - {1\over |\varvec{r}+{\varvec{R}/2}|} \end{aligned}$$

was expanded in spherical harmonics. Using this expansion, Eq. (13) of the wave-function, and expressing all other operators in Eqs. (12) and (15) as irreducible tensor operators^16^, one obtains a system of coupled equations for the functions $f_{lm}(r,t)$ in Eq. (13). This system was solved using the Lanczos algorithm^28^ (we used propagation stepsize $\Delta t=0.05$ a.u., and propagation algorithm of the order $N=30$). The initial ground state of the H$_2^+$ was prepared using the relaxation procedure^29^, propagating variational estimate for the initial wave-function in the imaginary time. Differential ionization probabilities $P(\varvec{p})$ are calculated by letting the wave-function of the system evolve for some time after the end of the pulse so that the wave-packet describing ionized electron state leaves the region where $r<R_0$ with some $R_0$ (we use the value $R_0=80$ a.u. in the calculations). In the region $r>R_0$ the true scattering states of H$_2^+$ can be well approximated by the single-center Coulomb scattering states, and to calculate the differential ionization probabilities we can project the TDSE solution on the ingoing Coulomb scattering states, calculating all the the radial integrals starting from $r=R_0$. This procedure proposed in^30^ helps to avoid possible non-orthogonality of molecular states belonging to bound and continuous spectra which is difficult to avoid in numerical calculations^31^. We checked that results we obtain are stable against further increase of the cutoff parameter $R_0$.

### Details of the tight binding model

A general expression for the amplitude we obtained was:

16 $$\begin{aligned} a_{\varvec{p}}= a^{(0)}_{\varvec{p}} + a^{(1)}_{\varvec{p}} \ , \end{aligned}$$

with

17 $$\begin{aligned} a^{(0)}_{\varvec{p}}= -i a(\Omega ) \langle \phi ^-_{\varvec{p}}|\hat{p}_z|\phi _0 \rangle \, \end{aligned}$$

and

18 $$\begin{aligned} a^{(1)}_{\varvec{p}} \approx \Omega c^{-1}a(\Omega ) \langle \phi ^-_{\varvec{p}}| \hat{p}_z x|\phi _0 \rangle \ , \end{aligned}$$

where $\Omega = E_{\varvec{p}}- \varepsilon _0$, and $a(\Omega )$, and $b(\Omega )$ are Fourier transforms of *A*(*t*) and $A^2(t)$. These expressions can be simplified if we assume first that the momentum is large enough so we can replace the scattering state $\phi ^-_{\varvec{p}}$ with the plane wave, and second, if we use a tight binding description for the bound state of H$_2^+$, i.e., we assume initial state wave-function can be represented as $\phi _0(\varvec{r}) =(\phi (\varvec{r}-{\varvec{R}/2}) + \phi (\varvec{r}+{\varvec{R}/2}))/\sqrt{2}$, where support of $\phi (\varvec{r})$ is some (sufficiently small) ball centered at the origin. We assume that $\phi (\varvec{r})$ is spherically symmetric, i.e. describes an *s*-state. It is easy to see that under these approximations:

19 $$\begin{aligned} a^{(0)}_{\varvec{p}}= a_d(\varvec{p}) \sqrt{2} \cos {\left( {\varvec{p}\cdot {\varvec{R}}\over 2}\right) } \ , \end{aligned}$$

and

20 $$\begin{aligned} a^{(1)}_{\varvec{p}}= -{\Omega \over c} {\partial \over \partial p_x} a^{(0)}_{\varvec{p}} \ . \end{aligned}$$

In Eq. (19):

21 $$\begin{aligned} a_d(\varvec{p})= -i a(\Omega ) \langle \varvec{p}|\hat{p}_z\phi \rangle \ \end{aligned}$$

is the dipole amplitude which we would obtain if electrons were emitted from the single-center initial wave-function $\phi (\varvec{r})$ centered at the origin.

From Eqs. (16), (19), and (20) we obtain:

22 $$\begin{aligned} a_{\varvec{p}}= a^{(0)}_{\varvec{p}} + a^{(1)}_{\varvec{p}} \approx a_d(\varvec{p}) \sqrt{2} \cos {\left( {\varvec{p}\cdot {\varvec{R}}\over 2} +\delta \right) } \ , \end{aligned}$$

where $$\displaystyle \delta =- {\Omega R_x\over 2c}$$.

### Origin of the Gaussian factor in Eq. (10)

Derivation of the Eq. (10) was based on the assumption that the dipole amplitude $a_d(\varvec{p})$ in the Eq. (7) can be represented as a a product of two factors, the factor $p_z$ responsible for the angular dependence of the amplitude and the Gaussian factor $\exp {\left\{ -b(p-p_0)^2\right\} }$, peaked around the expressing energy conserving momentum $p_0^2/2= \varepsilon _0+ \omega$. Possibility of such a representation can be seen if we examine a perturbative expression for $a_d(\varvec{p})$, which looks quite similar to the Eq. (4) above, with the difference that we should use a single-center wave-function $\phi (\varvec{r})$ to calculate the matrix element:

23 $$\begin{aligned} a_d(\varvec{p})= -i a(\Omega ) \langle \phi ^-_{\varvec{p}}|\hat{p}_z|\phi \rangle \ \ , \end{aligned}$$

where $\Omega = E_{\varvec{p}}- \varepsilon _0$, and $a(\Omega )$ is the Fourier transform of the pulse vector potential. Assuming that the scattering state in Eq. (23) can be approximated by a plane wave, and assuming for the moment Gaussian shape $A(t)=A_0e^{-\beta t^2}\sin {\omega t}$ for the pulse vector potential we obtain from Eq. (23):

24 $$a_d(\varvec{p})= {\mathrm{const}} \times {\tilde{\phi }}(\varvec{p}) p_z \exp {\left\{ -{(\Omega -\omega )^2\over 4\beta }\right\} }$$

where $\phi (\varvec{p})$ is Fourier transform of the single-center wave-function $\phi (\varvec{r})$. We do not write explicitly unimportant constant factors in Eq. (24), and we omitted in Eq. (24) exponentially small term proportional to $\displaystyle \exp {\left\{ -{(\Omega +\omega )^2\over 4\beta }\right\} }$. For long enough pulse (small value for the $\beta$-parameter) exponential factor in Eq. (23) is sharply peaked about the energy conserving value of $E_{\varvec{p}}$, and we can neglect the much slower momentum dependence of the Fourier transform of $\phi (\varvec{r})$ (since $\phi (\varvec{r})$ is assumed to represent an *s*-state its Fourier image $\tilde{\phi }(\varvec{p})$ depends only on $|\varvec{p}|$). For the same reason we can rewrite the exponential factor in Eq. (24) as: $\displaystyle \exp {\left\{ -{(\Omega -\omega )^2\over 4\beta }\right\} } =g(p)\exp {\left\{ -{p_0^2(p-p_0)^2\over 2\beta }\right\} }$ with $p_0$ such that $p_0^2/2= \varepsilon _0+ \omega$, is the energy conserving value of momentum, and *g*(*p*) is a function which varies much slower than the exponential factor in the region where the total expression is non-negligible, so that we can neglect its momentum dependence. That gives us the expression for the amplitude $a_d(\varvec{p})$ we used above. In the present calculation we used the pulse shape (14) different from the Gaussian shape, but one can see that near the photo-electron distribution maximum (*p* close to $p_0$) we can still use the Gaussian shape for the amplitude (this corresponds again to assuming that we neglect *p*-dependence of all slowly varying functions near $p=p_0$).

### Derivation of Eq. (9)

To derive Eq. (9) we substitute expression (10) for the amplitude into Eq. (8), and assume that at large distances, which interest us, the scattering states can be approximated by the plane waves. As we mentioned above, this assumption is not true if the Coulomb interaction is present. We make it only to simplify the derivation. The Coulomb interaction would add slowly varying logarithmic corrections which could easily be included into consideration. These Coulomb corrections, however, are not important for us as long as we are interested in the time delay difference between the wave packets. Representing the dipole amplitude as $a_d(\varvec{p})=|a_d(\varvec{p})|e^{i\eta }$, we obtain then the following integral for the wave-function describing ionized wave-packet (ignoring the unimportant constant factors):

25 $$\begin{aligned} \Psi _{\mathrm{ion}}(\varvec{r},t) = \mathrm{const}\times \int |a_d(\varvec{p})|\left( e^{iS_1(\varvec{p},\varvec{r},t)} +e^{iS_2(\varvec{p},\varvec{r},t)}\right) \ d\varvec{p}= I_1 + I_2\ , \end{aligned}$$

where

26 $$\begin{aligned} S_1(\varvec{p},\varvec{r},t)= & {} \eta +\varvec{p}\cdot \left( \varvec{r}+{{\varvec{R}}\over 2}\right) +\delta - {p^2t\over 2} \nonumber \\ S_2(\varvec{p},\varvec{r},t)= & {} \eta +\varvec{p}\cdot \left( \varvec{r}-{{\varvec{R}}\over 2}\right) -\delta - {p^2t\over 2} \end{aligned}$$

and we use notation $I_1$, $I_2$ for the integrals with the exponential functions $e^{iS_1(\varvec{p},\varvec{r},t)}$ and $e^{iS_1(\varvec{p},\varvec{r},t)}$, correspondingly. In Eq. (26) $\eta$ is the phase of the dipole amplitude $a_d(\varvec{p})$ and $\delta =-{\varvec{\kappa }}\cdot {\varvec{R}}/2$- is the phase due to the non-dipole effects from Eq. (7).

For large *t* the integrals in Eq. (25) can be computed using the saddle point method. Computing derivatives with respect to $\varvec{p}$ and taking into account that both $\delta$ and $\eta$ (for the case of the single-center wave-function $\phi (\varvec{r})$ describing an *s*-state) are functions of $|\varvec{p}|$, we can write the saddle point equation for the integral $I_1$ as follows:

27 $$\begin{aligned} \varvec{r}- \varvec{p}_{\mathrm{sp}}( t - \tau - \tau _1) + {{\varvec{R}}\over 2}=0 \ , \end{aligned}$$

where $\varvec{p}_{\mathrm{sp}}$ is the saddle point momentum, $\displaystyle \tau = {\partial \eta / \partial E}$ is the Wigner photoemission time-delay^20^ and $\tau _1= {\partial \delta / \partial E}= -R_x/(2c)$ is the time delay due to the relativistic non-dipole effects. Then, for $t\rightarrow \infty$ we obtain for the integral $I_1$:

28 $$\begin{aligned} I_1 \simeq {\mathrm{const}\over t^{3\over 2} } \times a_d(\varvec{p}_{\mathrm{sp}}) \exp {\left\{ i{\left( \varvec{r}+{{\varvec{R}}\over 2}\right) ^2 ({t\over 2}-\tau - \tau _1)\over (t-\tau -\tau _1)^2}+i\delta \right\} }\ , \end{aligned}$$

where $\varvec{p}_{\mathrm{sp}}$ is the root of the saddle-point equation (27). As we mentioned above, the dipole amplitude $a_d(\varvec{p})$ is essentially a product of two factors, the factor $p_z$ giving the angular dependence of the amplitude and the factor depending on the momentum magnitude $|\varvec{p}|$, which is peaked around the momentum value $p=p_0$ satisfying the energy conservation $p_0^2/2= \varepsilon _0+ \omega$. We can write therefore, using the expression (27) for $\varvec{p}_{\mathrm{sp}}$:

29 $$\begin{aligned} I_1 \simeq \left( z+{R_z\over 2}\right) \exp {\left\{ i{\left( \varvec{r}+{{\varvec{R}}\over 2}\right) ^2 ({t\over 2}-\tau - \tau _1) \over (t - \tau - \tau _1)^2} +i\delta \right\} } G\left( \left| \varvec{r}+{{\varvec{R}}\over 2}\right| -p_0(t-\tau - \tau _1)\right) \ , \end{aligned}$$

where we absorbed all the constant factors in the definition of the function *G*. Basing on the properties of the $a_d(\varvec{p})$ we discussed above, we may conclude that the function *G*(*u*) in Eq. (29) is sharply peaked around the value $u=0$. It describes, therefore, a wave-packet propagating from the point $\varvec{r}_0=-{\varvec{R}}/2$ and delayed by the amount $\tau + \tau _1$. For the integral $I_2$ in Eq. (25) we obtain quite analogously:

30 $$\begin{aligned} I_2 \simeq \left( z-{R_z\over 2}\right) \exp {\left\{ i{\left( \varvec{r}-{{\varvec{R}}\over 2}\right) ^2 ({t\over 2}-\tau + \tau _1) \over (t - \tau + \tau _1)^2} -i\delta \right\} } G\left( \left| \varvec{r}-{{\varvec{R}}\over 2}\right| -p_0(t-\tau + \tau _1)\right) \end{aligned}$$

with similar interpretation. Adding contributions (29) and (30) we obtain Eq. (9).
